# Four New Species and a New Record of *Panaeolus* from China, with Notes on the Taxonomy of *Panaeolus rhombispermus*

**DOI:** 10.3390/jof12030182

**Published:** 2026-03-03

**Authors:** Hong Cheng, Tolgor Bau

**Affiliations:** Key Laboratory of Edible Fungal Resources and Utilization (North), Ministry of Agriculture and Rural Affairs, Jilin Agricultural University, Changchun 130118, China; hongcheng156@outlook.com

**Keywords:** Galeropsidaceae, *Panaeolus*, *Crucispora*, new species, phylogeny, taxonomy

## Abstract

*Panaeolus* is a genus of small, dark-sporeda agarics within the family Galeropsidaceae. Based on the majority of specimens collected from China, this study investigated the genus *Panaeolus* and identified 17 species. These include four new species: *Panaeolus bambusicola*, *Panaeolus latifolius*, *Panaeolus praecox*, and *Panaeolus ovinus*; and one new record for China: *Panaeolus fraxinophilus*. The new species and the newly recorded species for China are morphologically described and illustrated. A multi-locus phylogenetic analysis (ITS, nrLSU, tef1-α, rpb2) was conducted using maximum likelihood and Bayesian inference. Combined morphological and phylogenetic evidence supports the reduction in the genus *Crucispora* to a subgenus within *Panaeolus*, accommodating *P. rhombispermus*.

## 1. Introduction

The genus *Panaeolus* (Fr.) Quél. was originally proposed as a subgenus of *Agaricus* L. by Fries (1836) [1], and subsequently unequivocally established at the generic level by Quélet (1872) [2]. As saprotrophic fungi, species of *Panaeolus* are globally distributed across temperate, tropical, and desert regions, typically inhabiting grasslands, herbivore dung, sandy soils, and wood chips. According to the Index Fungorum database (https://www.indexfungorum.org, accessed on 24 February 2026), the genus encompasses 203 names, including 45 infraspecific taxa. Of the approximately 158 remaining names at the species level, several have been reassigned to other genera, such as *Psathyrella* (Fr.) Quél. and *Psilocybe* (Fr.) P. Kumm. However, within modern taxonomic frameworks, likely fewer than half are currently accepted as distinct species. Notably, several species within *Panaeolus* are known to contain psilocybin and other related psychoactive compounds, the ingestion of which can lead to poisoning characterized by hallucinogenic effects [3,4,5].

The taxonomic history of the genus *Panaeolus* is marked by considerable debate, a consequence of the divergent methodological approaches and observational focuses employed by researchers over time. The concept of Panaeoloideae Singer was first proposed by Singer as a subfamily under Coprinaceae Overeem & Weese, encompassing *Panaeolina* Maire, *Panaeolus* (Fr.) Quél., *Copelandia* Bres., and *Anellaria* P. Karst., with *Panaeolus* (Fr.) Quél. as the type genus. This subfamily is distinguished from other subfamilies within the Coprinaceae primarily by a key microscopic character: its spores do not exhibit discolouration when treated with concentrated sulfuric acid (H_2_SO_4_). In contrast, Oláh (1969) [6] reclassified the genus within Strophariaceae Singer & A.H. Sm., consolidating related species into a single generic name *Panaeolus* based primarily on chemical and cultural characteristics. Singer (1976) challenged Oláh’s conclusions, critiquing the methodology, biassed character selection overemphasizing chemical traits, and the omission of key morphological features such as spore print color, solubility of spore pigments, and epicutis structure [7].

This historical debate illustrates the limitations of morphology-based classification and has been largely addressed by modern molecular phylogenetics. Molecular phylogenetic analyses have generated convergent insights. Tóth et al. (2013) demonstrated that the type species of *Galeropsis* Velen. (*Galeropsis desertorum* Velen. & Dvořák) clusters within the *Panaeolus*–*Panaeolina* clade [8]. Subsequently, based on examinations of type specimens and molecular phylogenetic analyses, Malysheva et al. (2019) formally combined the type species of *Galeropsis*, *G. desertorum*, into *Panaeolus* [9]. In a review that synthesized these findings, Kalichman et al. (2020) [10] further examined the taxonomic position of *Panaeolus* and suggested that Galeropsidaceae Singer should be recognized as the correct familial designation for the clade historically treated as tribe Panaeoleae or subfamily Panaeoloideae Singer, positing it as the proper name for the family containing *Copelandia* Bres., *Panaeolina* Maire, *Panaeolopsis* Singer, and *Panaeolus* (Fr.) Quél., with the proviso that this grouping continues to be supported as distinct from Bolbitiaceae Singer. More recently, this hypothesis was substantiated by He et al. (2026), who revised the taxonomic framework of Galeropsidaceae and, based on phylogenetic evidence, proposed a division of the genus *Panaeolus* into the following three subgenera: *Panaeolus* subg. *Bresadolomyces*, subg. *Panaeolina*, and subg. *Panaeolus* [11].

The taxonomic uncertainty extends to morphologically distinctive genera historically associated with *Panaeolus*. The genus *Crucispora* E. Horak was established in 1971 with *Crucispora naucorioides* E. Horak designated as its type species. However, its unique morphological characteristics, particularly the distinctive cruciform spores, precluded a clear familial assignment at the time [12]. Upon re-examining the type material, Singer acknowledged that its phylogenetic affinities remained unresolved but tentatively placed it within the Agaricaceae Chevall, based on a synthesis of morphological traits [7]. The species *Panaeolina rhombispermus* Hongo, originally described by Hongo (1973) [13], was subsequently transferred to *Crucispora* as *Crucispora rhombisperma* (Hongo) E. Horak, becoming the second species in the genus. The taxonomic status of this species remained uncertain for decades. Previous work by Ostuni et al. (2025) re-evaluated *C. rhombisperma* based on ITS and 28S phylogenetic data [14]. Their analysis revealed its close affinity with *P. mexicanus*, leading to the proposal of the new combination *Panaeolus rhombispermus* (Hongo) Birkebak, Voto & Ostuni. However, this conclusion was drawn mainly from molecular phylogenetic data. Therefore, the present study aims to re-evaluate the taxonomic status of *P. rhombispermus* by integrating novel morphological evidence from scanning electron microscopy (SEM) of spore morphology with multilocus phylogenetic analyses.

Previous records indicate over 30 species of *Panaeolus* in China [11,15,16,17,18,19,20], though some lack verifiable voucher specimens. Here, we report the findings from a four-year nationwide survey that yielded over 200 specimens. Using a combined morphological and phylogenetic approach, we identified 17 species, including four new species and one new record for China, which are described and illustrated in detail.

## 2. Materials and Methods

### 2.1. Sampling and Morphological Analyses

**Figure 1:**
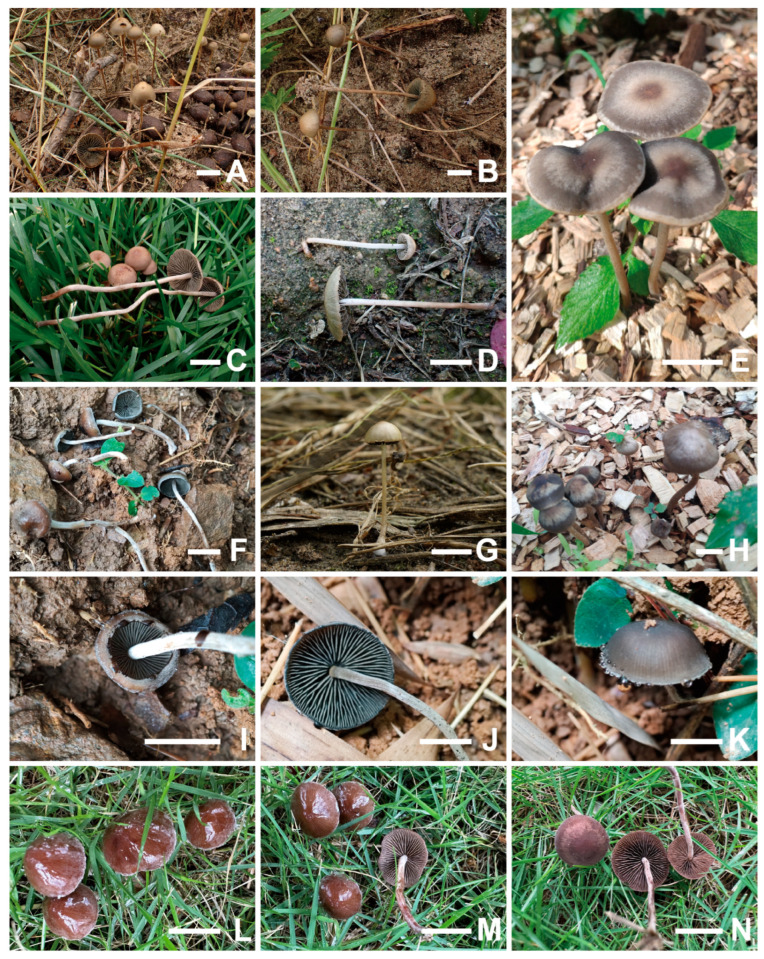
Basidiomata of *Panaeolus* species. (**A**,**B**) *Panaeolus ovinus*, (**C**) *Panaeolus praecox*, (**D**,**G**) *Panaeolus latifolius*, (**E**,**H**) *Panaeolus fraxinophilus*, (**F**,**I**–**K**) *Panaeolus bambusicola*, (**L**–**N**) *Panaeolus rhombispermus*. Scale bars = 1 cm.

Specimens for this study were collected from different regions in China, including Inner Mongolia Autonomous Region, Xinjiang Uygur Autonomous Region and the provinces of Jilin, Zhejiang, Guangdong, and Yunnan. Habitat photographs were captured in the field (Figure 1), and specimens were deposited at the Fungarium of Jilin Agricultural University (FJAU). We described the colours of all major morphological structures (e.g., fresh basidiocarps, lamellae, and spores) based on the Kornerup and Wanscher (1978) colour system [21]. The tissues of the specimens were treated with 5% KOH [22]. Microscopic observations were carried out with the aid of light microscopes (Carl Zeiss Primo Star, Jena, Germany; Olympus CX33, Tokyo, Japan). The basidiospore measurements do not include the apiculus and are presented as “(a)b–c(d) × e–f × g–h”, where “b–c” represents the minimum of 90% of the measured values and “a” and “d” represent the extreme values. Due to the dorsiventral flattening of *Panaeolus* basidiospores, their dimensions are view-specific: “e–f” represents the width in frontal view, and “g–h” the thickness in side view. The main body (sterigmata not included) of the basidia, cheilocystidia, pleurocystidia, caulocystidia, pileocystidia and pileipellis were measured (if present). The notation (n/m/p) indicates that measurements were made on “n” randomly selected basidiospores from “m” basidiomes of “p” collections. Q is the ratio of length divided by width, and Qm represents the average quotient (length/width ratio) standard deviation.

For scanning electron microscopy (SEM) analysis, the lamellae of air-dried samples were first mounted on specimen stubs using double-sided conductive adhesive tape. Subsequently, the samples were sputter-coated with gold using an IXRF MSP-2S ion sputter coater (IXRF Systems, Austin, TX, USA) and then observed under a Zeiss field-emission scanning electron microscope (Carl Zeiss AG, Oberkochen, Germany).

### 2.2. DNA Extraction, PCR Amplification, and Sequencing

Genomic DNA was extracted from the dried specimens using a NuClean Plant Genomic DNA kit (ComWin Biotech, CW0531M, Taizhou, China) according to the manufacturer’s instructions. The primer pairs ITS1F/ITS4 [23,24], LR0R/LR7 [25], EF1-983F/EF1-2212R [26], and RPB2-6F/RPB2-7.1R [27] were used to amplify the ITS, nrLSU, tef1-α, and rpb2 sequences, respectively. PCR amplifications were performed in 25 µL reactions containing: 2.0 µL template DNA, 12.5 µL 2× Es Taq Master Mix (Dye, ComWin Biotech, CW0690H, Taizhou, China), 1.0 µL of each primer (10 pmol/µL), and 8.5 µL of ddH2O. PCR products were purified and sequenced by Sangon Biotech (Shanghai, China).

### 2.3. Phylogenetic Analyses

Newly generated sequences were deposited in the NCBI GenBank database, while other sequences were retrieved from NCBI (accession numbers are provided in Table 1). The ITS, nrLSU, tef1-α, and rpb2 sequences were aligned separately using the online MAFFT v7 service with default parameters (https://mafft.cbrc.jp/alignment/server/, accessed on 31 March 2025) and subsequently refined manually in MEGA7 [28]. The resulting individual alignments were then concatenated into a multi-locus dataset using PhyloSuite v2 [29]. The final concatenated dataset (ITS + nrLSU + tef1-α + rpb2) consisted of aligned sequences with lengths of 743 bp (ITS), 1351 bp (nrLSU), 1191 bp (tef1-α), and 731 bp (rpb2), resulting in a total concatenated length of 4016 bp, with missing data coded as “?”. The best-fit partition and substitution models were selected using ModelFinder v2.2.0 [30]. For the maximum likelihood (ML) analysis, the best-fit models selected under the Bayesian Information Criterion (BIC) were TPM2u + F + G4 for the ITS dataset, TIM3 + F + R2 for the nrLSU dataset, SYM + I + G4 for the tef1-α dataset, and TNe + I + G4 for the rpb2 dataset. For the Bayesian inference (BI) analysis, the best-fit models selected under the Akaike Information Criterion (AIC) were GTR + F + I + G4 for the ITS dataset, GTR + F + I + G4 for the nrLSU dataset, SYM + I + G4 for the tef1-α dataset, and SYM + I + G4 for the rpb2 dataset. The ML analysis was performed with IQ-TREE 3 under the selected models, with branch support assessed based on 1000 standard bootstrap replicates [31]. Bayesian inference was conducted with MrBayes v3.2.7a [32] under the partition model. Four independent Markov Chain Monte Carlo (MCMC) chains were run for 10,000,000 generations, sampling trees every 1000 generations. The first 25% of sampled trees were discarded as burn-in after the average standard deviation of split frequencies dropped below 0.01. Resulting consensus trees were visualized in FigTree v1.4.4 and prepared for publication using Adobe Illustrator 2021.

## 3. Results

### 3.1. Molecular Phylogeny

**Figure 2:**
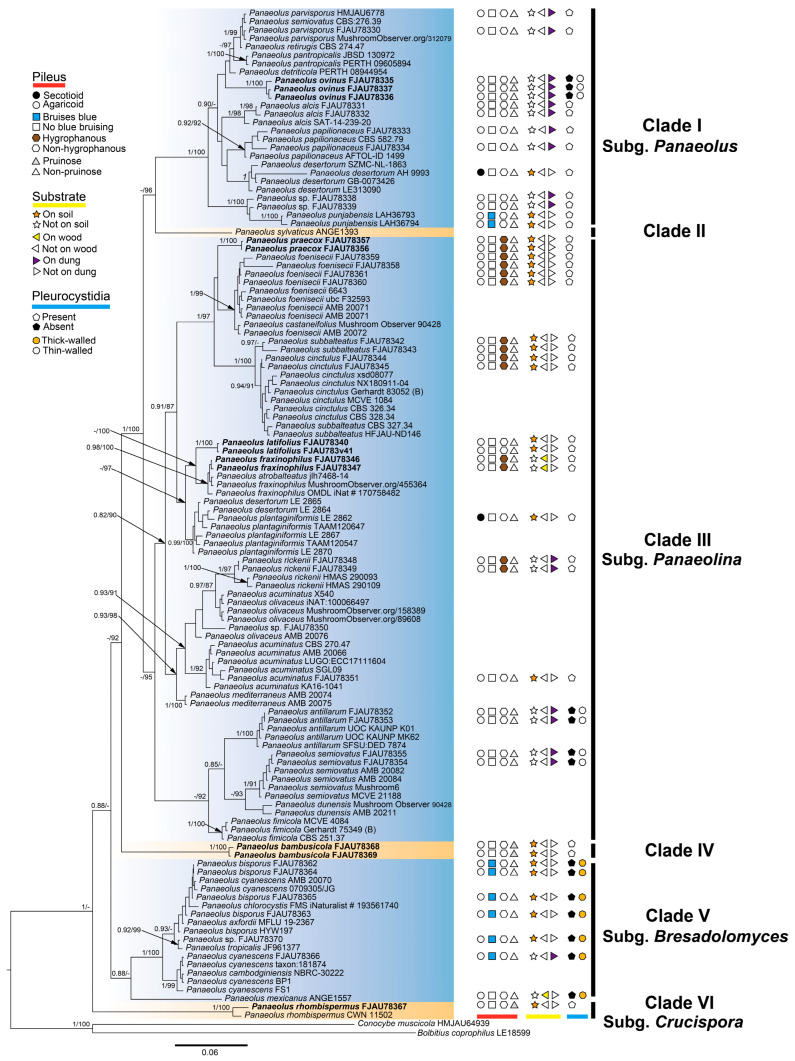
Phylogeny of *Panaeolus* inferred from Bayesian and maximum-likelihood analyses of a multi-locus dataset (ITS, nrLSU, tef1-α, and rpb2). Nodal support values are shown. Key morphological features (pileus, pleurocystidia) and substrate preferences are summarized in the right panel.

This study generated 95 new sequences (ITS: 40, nrLSU: 21, tef1-α: 18, and rpb2: 16), which were combined with 120 sequences retrieved from GenBank, resulting in a final dataset of 120 taxa. *Conocybe muscicola* and *Bolbitius coprophilus* (Bolbitiaceae) were selected as the outgroup. The Bayesian inference (BI) and maximum likelihood (ML) analyses yielded largely congruent tree topologies. Only the BI tree is presented (Figure 2), with nodal support indicated as Bayesian posterior probabilities (PPs) and ML bootstrap values (MLbs); nodes with PP ≥ 0.80 and MLbs ≥ 80% are shown.

The phylogenetic reconstruction did not support the traditional generic boundaries among *Panaeolina*, *Copelandia*, *Anellaria*, and *Panaeolus* sensu stricto, further supporting their consolidation into a single, unified genus. Within *Panaeolus*, six major clades (I–VI) were identified, each highlighted with a distinct background colour in Figure 2.

*Panaeolus ovinus* (vouchers: FJAU78335–37) was placed within Clade I (subg. *Panaeolus*) and is distinguished by the presence of pleurocystidia, a trait not observed in other members of this subgenus. Within Clade III (subg. *Panaeolina*), the following three taxa were of particular interest: (1) *P. praecox* (vouchers FJAU78356–57) was a sister to *P. foenisecii*. These species are morphologically distinguished by spore ornamentation (*P. foenisecii*: verrucose; *P. praecox*: smooth) but share an exclusive occurrence on grassy lawns and hygrophanous pilei. (2) *P. latifolius* (vouchers FJAU78340–41) and (3) the newly recorded *P. fraxinophilus* (vouchers FJAU78346–47) formed a sister-group relationship. While sharing smooth-walled basidiospores, they differ in substrate preference and pileus characteristics: *P. latifolius* occurs on sandy soil and lacks hygrophany, whereas *P. fraxinophilus* grows on wood chips and exhibits a distinctly hygrophanous pileus. *P. bambusicola* (vouchers FJAU78368–69) formed a distinct lineage, which is here designated as Clade IV. This species is unique among known *Panaeolus* taxa in its exclusive bamboo forest habitat and is further characterized by a persistently pruinose pileus, abundant pileocystidia, and relatively small basidiospores.

Notably, *P. rhombispermus* (vouchers: FJAU78367, CWN11502) formed an independent lineage identified as Clade VI (subg. *Crucispora*). This phylogenetic placement is corroborated by its distinctive cruciform basidiospores. Consequently, we treat this lineage as a distinct subgenus within the genus *Panaeolus*.

### 3.2. Taxonomy

***Panaeolus ovinus*** T. Bau & H. Cheng, **sp. nov.**

Mycobank No.: MB861990

**Figure 3:**
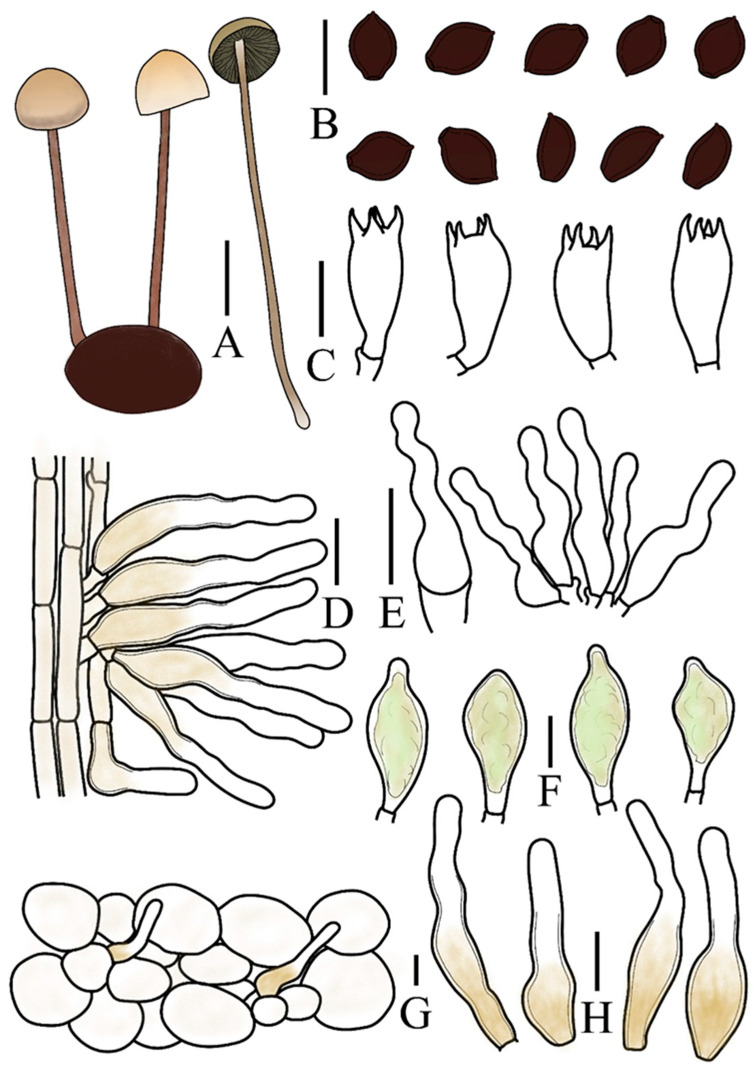
*Panaeolus ovinus* (FJAU78336) (**A**) basidiomata, (**B**) basidiospores, (**C**) basidia, (**D**) caulocystidia, (**E**) cheilocystidia, (**F**) pleurocystidia, (**G**) pileipellis element (**H**) pileocystidia. (**A**) Scale bar = 1 cm; (**B**–**H**) scale bars = 10 μm.

Figure 1A,B and Figure 3

Etymology. “ovinus” (Latin, pertaining to sheep) refers to its growth on sheep dung.

Holotypus. CHINA. Jilin Province, Baicheng City, Tongyu County, Xianghai National Nature Reserve, 25 August 2023, 122°20′23″ E, 45°02′39″ N, alt. 137 m, Liyang Zhu, Z2382506 (FJAU78336).

Diagnosis: *Panaeolus ovinus* is distinguished from other coprophilous *Panaeolus* species by its relatively small, non-bruising blue basidiomata. It is macroscopically similar to *P. alcis* M.M. Moser but differs in having pleurocystidia.

Pileus 0.5–1.5 cm in diameter, campanulate, paraboloid, or hemispherical, margin weakly hygrophanous, centre blond (4C4), transitioning to greyish yellow (4B4) or pale yellow (4A4) toward the margin, drying brownish grey (5C2). Context thin, yellowish white (4A2) to grey (4B1), without a distinctive odour. Lamellae adnate to adnexed, moderately close, unequal, irregularly mottled with dark grey (1F1) or yellowish brown (5F8) to pale grey (1B1), edge even and remaining paler greyish. Stipe 4.0–6.5 cm long, 1.0–2.0 mm thick, cylindrical, slightly enlarged at the base, pale ochraceous grey, brownish toward the base, entirely pruinose, with the pruina more densely distributed on the middle and lower parts, and slightly longitudinally striated.

Spores (60/3/3) (7.8–)8.4–10.5(–11.5) × 6.1–8.2 × 5.0–6.5 µm, Q = 1.28–1.55, Qm = 1.39(0.07) in frontal view, limoniform to broadly limoniform, wall thick, smooth, Q = 1.55–1.82, Qm = 1.70(0.07) in side view, asymmetrical, pyriform, with a distinct germ pore, central, or slightly eccentric, spores appear dark brown (7F6) to black in a 5% KOH solution. Basidia (13–)16–23 × (7–)8–10 µm, nearly elliptical, clavate, 4-sterigmate, sterigmata 2–4 µm long. Cheilocystidia (15–)16–22(–25) × 3.5–5.5 µm, versiform, predominantly lageniform to narrowly utriform but with a ventricose base and an elongated, flexuose neck. Pleurocystidia (20–)23–27(–29) × (10–)11–12(–13) µm, thin-walled, functioning as chrysocystidia in KOH and containing yellow pigments, pedunculate or pedicellate, mostly with an obtuse apex. Caulocystidia (29–)32–40(–47) × (4.5–)5–7(–8.5) µm, versiform, predominantly narrowly cylindrical or less frequently geniculate, with both forms typically broadening toward the base, pale yellow (4A3) with slightly thickened walls on the medial and lower parts. Pileipellis hymeniform, composed predominantly of subglobose or globose elements, (15–)20–29(–40) × (14–)18–28(–36) µm, with yellow pigments at the base. Pileocystidia morphologically similar to caulocystidia, 32–46 × 8.5–10.5 µm. Clamp connections present.

Habitat. Gregarious to scattered on sheep dung in early autumn.

Known distribution. Currently, only known in Jilin Province, China.

Additional specimens measured. CHINA. Jilin Province, Baicheng City, Tongyu County, Xianghai National Nature Reserve, 25 August 2023, Hanbing Song, S2382511 (FJAU78337), Shien Wang, E2308343 (FJAU78335).

Notes. Although a considerable number of species in *Panaeolus* grow on dung, current records indicate that only *P. ovinus* is specifically associated with sheep dung. A morphologically similar species, *P. alcis* [41], can be distinguished from *P. ovinus* by its elongate-ellipsoid spores and the absence of pleurocystidia. The fruiting bodies of *P. papilionaceus* (Bull.) Quél. are generally markedly larger than those of *P. ovinus* and typically bear distinctive white, pyramidal veil remnants at the pileus margin. Under nutrient-deficient conditions, however, developmental constraints and the abrasion of these remnants by rain or wind may cause *P. papilionaceus* to closely resemble *P. ovinus*. Adding to this morphological convergence, both species share lemon-shaped spores in frontal view. The absence of pleurocystidia in *P. papilionaceus*, nevertheless, provides a stable and definitive diagnostic character for differentiation.

***Panaeolus praecox*** T. Bau & H. Cheng, **sp. nov.**

Mycobank No.: MB861989

**Figure 4:**
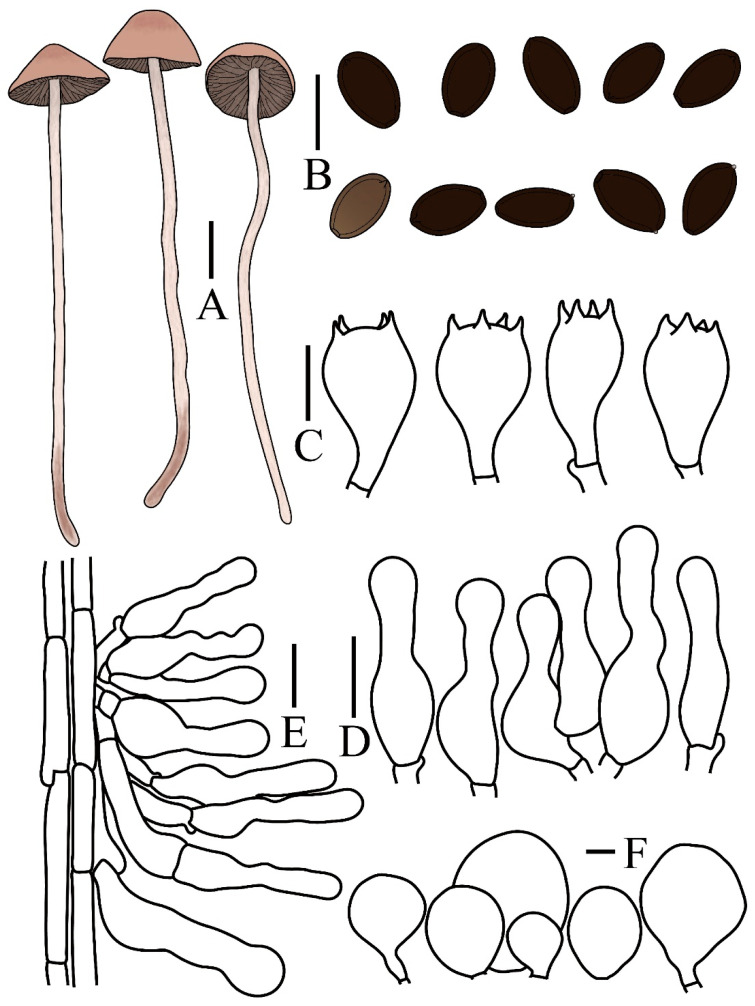
*Panaeolus praecox* (FJAU78357) (**A**) basidiomata, (**B**) basidiospores, (**C**) basidia, (**D**) cheilocystidia, (**E**) caulocystidia and (**F**) pileipellis element. (**A**) Scale bar = 1 cm; (**B**–**F**) scale bars = 10 μm.

Notes. *Panaeolus foenisecii* (Pers.) J. Schröt., *P. cinctulus* (Bolton) Sacc., and *P. oligotrophus* Ostuni & Voto are macroscopically similar to *P. praecox* and share comparable grassy habitats, all exhibiting a hygrophanous pileus. Spore morphology provides critical diagnostic characters for their differentiation. Only *P. foenisecii* possesses verrucose spores, whereas others are smooth. The spores of *P. cinctulus* are predominantly lemon-shaped, while those of *P. praecox* and *P. oligotrophus* are ellipsoid to oval; additionally, the spores of *P. praecox* are generally more elongated than those of *P. oligotrophus*. Based on current knowledge, P. praecox also fruits earlier in the growing season than other documented *Panaeolus* species within China. *P. subfoenisecii* M.Q. He & R.L. Zhao can be further distinguished by its slightly larger spores compared to *P. praecox* and the absence of caulocystidia [11].

Figure 1C and Figure 4

Etymology. “praecox” (Latin, early-maturing or precocious) refers to its characteristic of fruiting earlier in the season compared to other *Panaeolus* species found in grassy habitats.

Holotypus. CHINA. Jilin Province, Changchun City, Jilin Agricultural University campus, 9 July 2023, 125°24′14″ E, 43°48′35″ N, alt. 352 m, Hong Cheng, C2370902 (FJAU78357).

Diagnosis: *Panaeolus praecox* is distinguished by its earlier fruiting phenology compared to other grassy *Panaeolus* species, a hygrophanous pileus, and smooth-walled, ellipsoid to elongate-ellipsoid basidiospores in frontal view.

Pileus 1.5–2.5 cm in diameter, conical, broadly conical, or obtusely conical, margin hygrophanous, centre reddish brown (8D8), greyish red (8C5) to dull red (8C4), drying dull red (8B3) to reddish grey (8B2). Context thin, reddish white (8A2) to grey (8B1), without a distinctive odour. Lamellae adnate to adnexed, moderately close, unequal, irregularly mottled with dark grey (1F1) or brownish grey (8E2) to pale grey (1B1), edge smooth and remaining paler greyish. Stipe 7.0–9.0 cm long, 2.0–3.0 mm thick, cylindrical, erect to flexuous, slightly enlarged at the base, white (7A1) or pinkish (7A2), densely pruinose and slightly longitudinally striated, developing brownish discolorations where bruised or handled.

Spores (60/3/3) (9.5–)10.2–11.8(–14.0) × 6.2–7.5 × 5.3–6.5 µm, Q = 1.48–1.85, Qm = 1.63(0.09) in frontal view, ellipsoid to elongate-ellipsoid, wall thick, smooth, Q = 1.63–1.94, Qm = 1.80(0.09) in side view, elongate-ellipsoid, with a distinct germ pore, central, or slightly eccentric, spores appear dark brown (7F6) to black or brown (7E5) in a 5% KOH solution. Basidia (18–)19–22 × (10.5–)11–12.5 µm, clavate to broadly clavate, 4-sterigmate, sterigmata 2–4 µm long. Cheilocystidia (18–)19–24(–27) × 5.5–7.5 µm, versiform, predominantly narrowly utriform, often with a flexuous neck. Pleurocystidia absent. Caulocystidia (19–)23–33(–35) ×5.5–8.5(–9.2) µm, versiform, predominantly narrowly utriform with a flexuous neck to narrowly cylindrical. Pileipellis hymeniform, composed predominantly of subglobose or globose elements, (14–)28–33(–48) × (14–)26–32(–40) µm. Pileocystidia were not observed. Clamp connections present.

Habitat. Gregarious to scattered on lawns during spring or early summer.

Known distribution. Currently, this species has been recorded only in Jilin and Hunan Provinces, China.

Additional specimens measured. CHINA. Hunan Province, Changsha City, 16 April 2024, Changzhuo Liu, C2441601 (FJAU78356).

***Panaeolus latifolius*** T. Bau & H. Cheng, **sp. nov.**

Mycobank No.: MB861988

**Figure 5:**
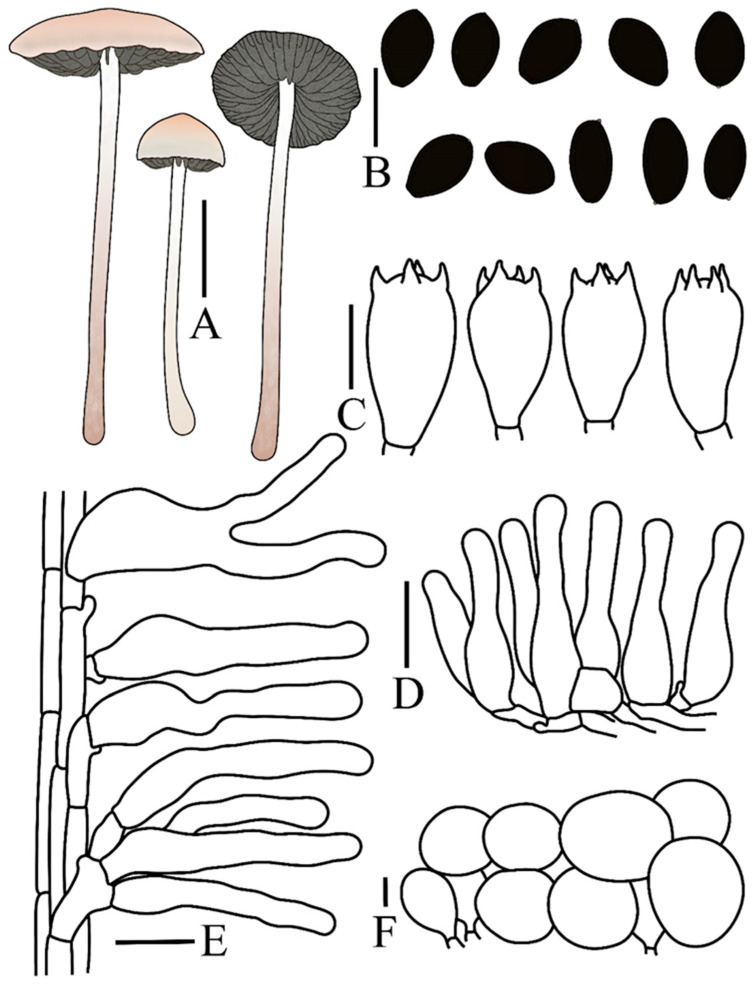
*Panaeolus latifolius* (FJAU78340) (**A**) basidiomata, (**B**) basidiospores, (**C**) basidia, (**D**) cheilocystidia, (**E**) caulocystidia and (**F**) pileipellis element. (**A**) Scale bar = 1 cm; (**B**–**F**) scale bars = 10 μm.

Figure 1D,G and Figure 5

Etymology. “latifolius” (Latin, broad-gilled) refers to its conspicuously wide lamellae that remain visible even in a frontal view of the basidioma.

Holotypus. CHINA. Inner Mongolia Autonomous Region, Tongliao City, Xar Moron Park, 19 July 2023, 122°15′28″ E, 43°37′55″ N alt. 260 m, Hong Cheng, C2371907 (FJAU78340).

Diagnosis: *Panaeolus latifolius* is distinguished by its convex to nearly applanate pileus, which causes the lamellae to appear conspicuously broad and ventricose. When the basidiomata are placed vertically and viewed frontally, the lamellae remain distinctly visible—a characteristic that distinguishes it from most other *Panaeolus* species. It further differs by its occurrence on sandy soil and non-hygrophanous pileus.

Pileus 0.8–1.8 cm in diameter, initially hemispherical, becoming convex to plano-convex at maturity, non-hygrophanous; centre greyish orange (5B4) to greyish red (7B6), gradually transitioning to orange white (5A2) or reddish grey (7B2) toward the margin. Context thin, reddish white (7A2) to grey (7B1), without distinctive odour. Lamellae adnate to adnexed, moderately close, unequal, distinctly ventricose, irregularly mottled with dark grey (1F1) to pale grey (1B1), edge even and remaining paler greyish. Stipe 2.0–4.5 cm long, 1.0–2.0 mm thick, cylindrical, erect, slightly enlarged at the base, white (7A1), pruinose and slightly longitudinally striated, or brownish toward the base.

Spores (60/3/3) (9.5–)11.0–12.3(–13.7) × 6.0–7.1 × 5.3–6.4 µm, Q = 1.45–1.80, Qm = 1.58 (0.09) in frontal view, ovoid, ellipsoid to elongate-ellipsoid, wall thick, smooth, Q = 1.65–1.93, Qm = 1.76(0.08) in side view, elongate-ellipsoid, with a distinct germ pore, central, or slightly eccentric, spores appear dark brown (7F6) to black in a 5% KOH solution. Basidia (14.5–)16–20(–23) × 8–10 µm, nearly elliptical to clavate, 4-sterigmate, sterigmata 2–4 µm long. Cheilocystidia (14–)17–25(–27) × 5.0–7.0 µm, predominantly narrowly lageniform to narrowly utriform. Pleurocystidia absent. Caulocystidia (23–)35–45 × 7.5–8.5(–12.8) µm, versiform, predominantly narrowly lageniform to narrowly utriform, occasionally furcate. Pileipellis hymeniform, composed predominantly of subglobose or globose elements, (22–)24–36(–41) × (20–)23–30(–40) µm. Pileocystidia were not observed. Clamp connections present.

Habitat. Scattered on sandy soil during summer and autumn.

Known distribution. Currently, this species is currently known only from Jilin Province and the Inner Mongolia Autonomous Region, China.

Additional specimens measured. Jilin Province, Baicheng City, Tongyu County, Xianghai National Nature Reserve, 26 August 2023, 122°20′10″ E, 44°50′39″ N, alt. 145 m, Liyang Zhu, Z2382617 (FJAU78341).

Notes. *Panaeolus latifolius* is characterized by its occurrence on sandy soil and small basidiomata with frontally visible lamellae. The species further exhibits a non-hygrophanous pileus that becomes convex to plano-convex at maturity, absence of pleurocystidia, and no blue bruising reaction. This unique combination of features allows clear differentiation from other *Panaeolus* species. *P. fraxinophilus* A.H. Sm., while also possessing a convex pileus, is distinguished by its fuscous-black pileus colour, hygrophanous condition when moist, and lignicolous habit. These pronounced macroscopic differences allow for reliable separation from *P. latifolius*.

***Panaeolus bambusicola*** T. Bau & H. Cheng, **sp. nov.**

Mycobank No.: MB861987

**Figure 6:**
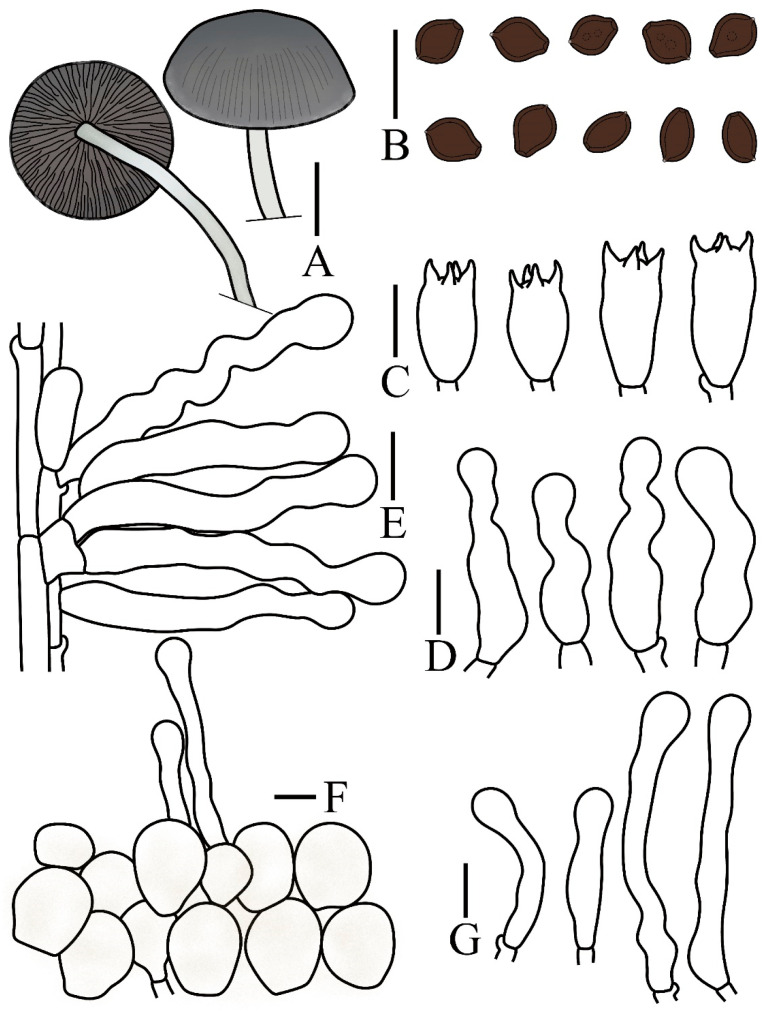
*Panaeolus bambusicola* (FJAU78368) (**A**) basidiomata, (**B**) basidiospores, (**C**) basidia, (**D**) cheilocystidia, (**E**) caulocystidia (**F**) pileipellis element (**G**) pileocystidia. (**A**) Scale bar = 1 cm; (**B**–**G**) scale bars = 10 μm.

Figure 1F,I–K and Figure 6

Etymology. “bambusicola” (Latin, from bambusa “bamboo” and -cola “inhabitant”) refers to its specific habitat in bamboo forests.

Holotypus. CHINA. Zhejiang Province, Huzhou City, Changxing County, 7 June 2024, 120°58′35″ E, 30°57′44″ N alt. 10 m, Zhuoluo Zhou, 1654 (FJAU78368).

Diagnosis: *Panaeolus bambusicola* is distinguished by its unique ecological habit of growing on bamboo forest soil, a pruinose pileus margin, absence of pleurocystidia, lack of a blue bruising reaction, and relatively small basidiospores measuring less than 10 µm in length.

Pileus 0.5–2.0 cm in diameter, initially campanulate, becoming broadly conical to convex at maturity, striate, pruinose at the margin; centre brown (7E8 to 7E3) or grey (7E8), transitioning to reddish grey (7B2) or pale grey (7C1) toward the margin. Context thin, without distinctive odour. Lamellae adnate to adnexed, moderately close, unequal, distinctly ventricose, irregularly mottled with dark grey (1F1) to pale grey (1B1), edge even and remaining paler greyish. Stipe 2.0–4.0 cm long, 1.0–2.0 mm thick, cylindrical, erect to flexuous, slightly enlarged at the base; surface white (7A1) to pale grey (1B1), becoming brownish toward the base, densely pruinose and faintly longitudinally striate, developing reddish brown discolorations where bruised or handled.

Spores (60/3/3) (6.0–)6.5–7 × 4.8–5.8 × 4.0–5.0 µm, Q = 1.10–1.40, Qm = 1.23 (0.06) in frontal view, broadly limoniform to nearly subglobose, wall thick, smooth, Q = 1.38–1.65, Qm = 1.53(0.09) in side view, ellipsoid, with a distinct germ pore, central, spores appear dark brown (7F6) to black in a 5% KOH solution. Basidia (12–)13–16(–17.5) × 7.2–8.5 µm, nearly elliptical to clavate, 4-sterigmate, sterigmata 2–4 µm long. Cheilocystidia (22–)24–30(–34) × 6.0–7.5 µm, flexuose with obtuse apex. Pleurocystidia absent. Caulocystidia (30–)45–60 × 7.0–9(–10.5) µm, flexuose with obtuse apex. Pileipellis hymeniform, composed predominantly of subglobose or globose elements, (13–)18–22(–24) × (12–)15–20(–21) µm. Pileocystidia nearly narrowly cylindrical but curved, or flexuose with an obtuse apex. Clamp connections present.

Habitat. Scattered on soil sections under bamboo forest during summer.

Known distribution. Currently, only known in Zhejiang Province, China.

Additional specimens measured. CHINA. Zhejiang Province, Huzhou City, Changxing County, 12 June 2025, 120°58′25″ E, 30°57′18″ N alt. 9 m, Zhuoluo Zhou, 1713 (FJAU78369).

Notes. *Panaeolus bambusicola* is characterized by its occurrence on soil in bamboo forests, a pruinose pileus margin, and relatively small spores. Although a pruinose pileus margin is also observed in *P. rhombispermus*, the latter is instantly distinguished by its unique cruciform basidiospores. This combination of ecological and micromorphological features reliably distinguishes *P. bambusicola* from all other known *Panaeolus* species.

***Panaeolus fraxinophilus*** A.H. Sm.

**Figure 7:**
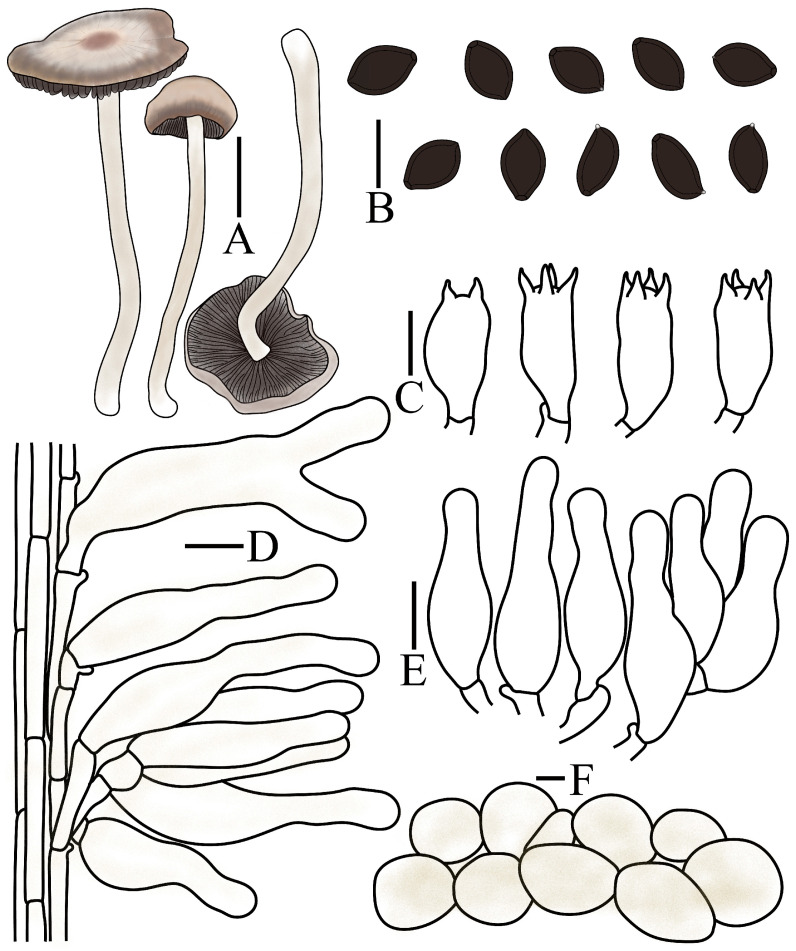
*Panaeolus fraxinophilus* (FJAU78346, FJAU78347) (**A**) basidiomata, (**B**) basidiospores, (**C**) basidia, (**D**) caulocystidia, (**E**) cheilocystidia and (**F**) pileipellis element. (**A**) Scale bar = 1 cm; (**B**–**F**) scale bars = 10 μm.

Figure 1E,H and Figure 7

Pileus 1–2.5 cm in diameter, initially campanulate, becoming convex to applanate or broadly conical with a subumbonate disc at maturity. Surface smooth or lacunose, hygrophanous when moist, with distinct striations; margin occasionally irregularly undulate, centre fuscous black (6F1 to 6F2), transitioning to grey (6D1) or pale grey (6C1) toward the margin, then darkening again to fuscous black near the edge, drying uniformly to grey (6D1). Context thin, without distinctive odour. Lamellae adnate, moderately close, unequal, irregularly mottled with dark grey (1F1) to pale grey (1B1), edge even and remaining paler greyish. Stipe 2.0–8.0 cm long, 2.0–3.0 mm thick, cylindrical, erect to flexuous, slightly enlarged at the base; surface pale grey (1B1), becoming brownish grey toward the base, densely pruinose and faintly longitudinally striate.

Spores (60/3/3) 9.0–10.5(–13.5) × 6.5–7.5(–8.0) × 5.0–6.3 µm, Q = 1.32–1.63, Qm = 1.50 (0.07) in frontal view, ovate, ellipsoid, wall thick, smooth, Q = 1.67–2.07, Qm = 1.87(0.11) in side view, elongate-ellipsoid, asymmetrical, with a distinct germ pore, eccentric, spores appear dark brown (7F6) to black in a 5% KOH solution. Basidia 19–21 × 7.8–9.5 µm, nearly elliptical to clavate, 4(2)-sterigmate, sterigmata 2–4 µm long. Cheilocystidia (24–)29–32(–35) × 9.0–11.2 µm, narrowly utriform. Pleurocystidia absent. Caulocystidia (37–)40–60 × 7.0–10.0(–12.5) µm, narrowly utriform, or with an elongated neck. Pileipellis hymeniform, composed predominantly of subglobose or globose elements, (25–)32–55(–65) × (24–)30–54(–60) µm. Pileocystidia absent. Clamp connections present.

Habitat. Gregarious to scattered on wood chip piles during spring.

Known distribution. Asia: China; North America: United States of America (Holotype), Canada; South America: Brazil.

Additional specimens measured. CHINA. Guangdong Province, Shenzhen City, 18 March 2024, Jianfeng Tan, C2431801(FJAU78346), Yunnan Province, Wenshan City, 18 May 2024, Xiangyang Li, C2452504 (FJAU78347).

Notes. Within the genus *Panaeolus*, lignicolous species are relatively uncommon. *Panaeolus atrobalteatus* Pegler & A. Henrici and *P. fraxinophilus* represent two such species that demonstrate remarkable morphological similarity. Both exhibit strongly hygrophanous pilei in moist conditions and possess closely overlapping spore size ranges [42]. It is noteworthy that in the protologue of *P. atrobalteatus*, Henrici did not undertake a comparative analysis with the morphologically similar *P. fraxinophilus*, nor was this taxon mentioned. Based on available herbarium material and the literature, the basidiomata of *P. atrobalteatus* are generally larger in overall dimensions than those of *P. fraxinophilus*, a characteristic that may provide a practical morphological criterion for their differentiation.

***Panaeolus*** **subg.** ***Crucispora*** (E. Horak) T. Bau & H. Cheng, **comb. and stat. nov.**

Mycobank No.: MB862146

Basionym. *Crucispora* E. Horak, New Zealand J. Bot. 9(3): 489 (1971)

Type species. *Panaeolus naucorioides* (E. Horak) T. Bau & H. Cheng

Description emended based on E. Horak’s (1971) [12] concept of *Crucispora*: Basidiomata small to medium-sized, mycenoid or naucorioid, not staining blue when injured. Pileus hemispherical to conical when young, becoming convex to campanulate; surface dry, hygrophanous, colour deep brown, tobacco-brown; margin non-striate or finely striate, bearing a white, pruinose coating (veil remnants) when young, disappearing with age. Context thin, odour and taste not distinctive. Lamellae adnate to adnexed, ventricose, edge white. Stipe cylindrical, fistulose; no persistent cortina or annulus. Basidiospores in deposit brown to dark brown or black, cruciform-rhomboid.

Notes. *Crucispora rhombispermus* (Hongo) was transferred to *Panaeolus* (Fr.) Quél. by Birkebak, Voto & Ostuni based on phylogenetic analyses of ITS and nrLSU sequences [14]. Subsequently, He et al. placed *P. rhombispermus* into *Panaeolus* subg. *Bresadolomyces* M.Q. He & R.L. Zhao, mainly based on phylogenetic evidence [11]. However, our results indicate that this species is clearly distinct from other members of subg. Bresadolomyces. Furthermore, the type species of *Crucispora*, *C. naucorioides* E. Horak, also possesses cruciform spores. Based on these findings, we propose to reduce the genus *Crucispora* to a subgenus within *Panaeolus*.

***Panaeolus naucorioides*** (E. Horak) T. Bau & H. Cheng, **comb. nov.**

Mycobank No.: MB862188

Basionym. *Crucispora naucorioides* E. Horak, New Zealand J. Bot. 9(3): 489 (1971)

Notes. According to E. Horak’s description of *Crucispora naucorioides*, features such as its hygrophanous pileus, buff-brown lamellae with white edges, adnate to adnexed attachment, and a hymeniform pileipellis closely resemble those of species in the genus *Panaeolus* [12]. Furthermore, its spores are cruciform, as in P. rhombispermus. Based on this morphological congruence, we propose to transfer this species into the genus *Panaeolus* and place it within the subgenus *Panaeolus* subg. *Crucispora*.

## 4. Discussion

Our integrated taxonomic reconstruction of *Panaeolus* delineates six major clades (I–VI) and describes four new species and one new record from China. Notably, based on phylogenetic and morphological evidence (particularly the cruciform basidiospores), we propose recognizing Clade VI (*P. rhombispermus*) as a distinct subgenus, *Panaeolus* subg. *Crucispora*. It should be noted, however, that the sampling localities in this study cover approximately 80% of China’s land area, spanning multiple provinces and autonomous regions. Specimens of *Panaeolus* from China’s Taiwan province, Hainan province, Qinghai province, and the Xizang Autonomous Region were not obtained; therefore, the diversity of the genus in these regions remains to be investigated in future studies. Furthermore, future discoveries of additional *Panaeolus* species worldwide may necessitate further refinement and revision of the taxonomic framework proposed here.

Among the newly described taxa, *Panaeolus ovinus* is currently the only known species in subg. *Panaeolus* that possesses pleurocystidia, with sheep dung as its sole known substrate. *P. praecox* is most closely related to *P. foenisecii* but is distinguished by its smooth spore surface. *P. latifolius* grows on sandy soil and, unlike the newly recorded species *P. fraxinophilus* (which grows on wood chips), we observed no hygrophany in the pileus of collected specimens—a characteristic that may be attributed to the species’ inherent traits or the relatively arid environment where it occurs. To our knowledge, *P. bambusicola* represents the first recorded species of *Panaeolus* found in bamboo forest habitats, characterized by relatively small spores and a frequently pruinose pileus. Correspondingly, this species exhibits abundant pileocystidia, whereas pileocystidia are generally sparse or even absent in other *Panaeolus* species. These findings enrich the known species diversity of *Panaeolus* in China and deepen our understanding of the habitat preferences and micromorphological characteristics within this genus.

In early taxonomy that relied predominantly on morphological characters, species of *Panaeolus* were historically classified into different genera or subgenera based on traits such as blue-bruising reaction upon injury, presence or absence of an annulus, spore ornamentation, and the morphology and occurrence of pleurocystidia [7]. In the present study, based on collected *Panaeolus* specimens and clear descriptions available in the literature, we summarized pileus characteristics, pleurocystidia types, and substrate preferences across the genus. The results indicate that these morphological features do not fully correspond to the six clades revealed by phylogenetic analysis, i.e., no shared derived characters (synapomorphies) useful for subdivision of the genus were identified. Because the phylogenetic framework obtained here is largely congruent with that of He et al. [11], we have adopted a similar subdivision of *Panaeolus*. However, in our phylogeny, *Panaeolus rhombispermus* (vouchers: FJAU78367, CWN 11502) form an independent lineage.

**Figure 8:**
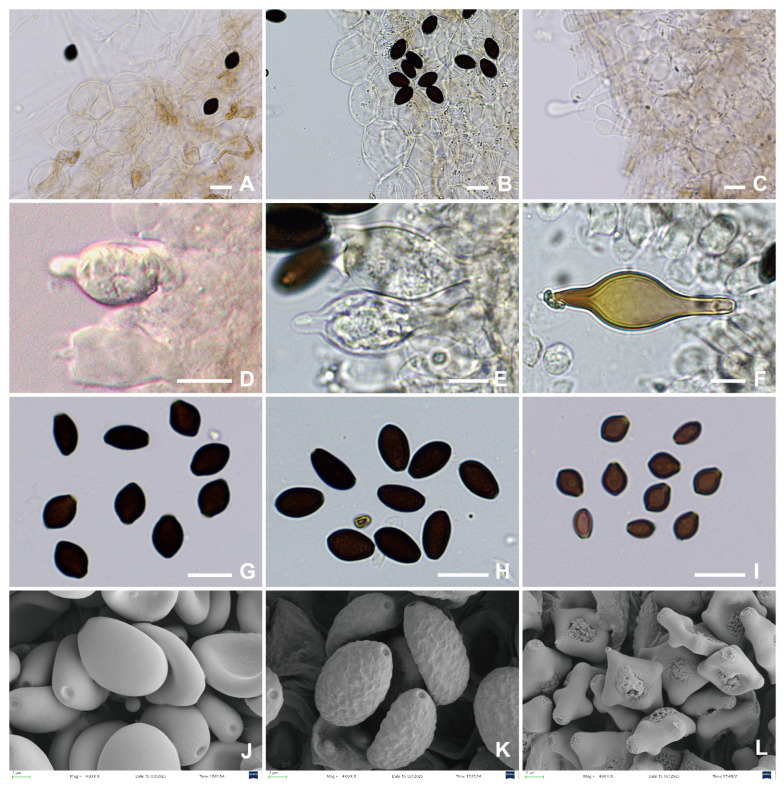
Micro-morphological structures of *Panaeolus* species. (**A**–**C**) Pileipellis: (**A**) *Panaeolus ovinus* (FJAU78336). (**B**) *P. latifolius* (FJAU78340). (**C**) *P. bambusicola* (FJAU78368); (**D**–**F**) Pleurocystidia: (**D**) *P. ovinus* (FJAU78336). (**E**) *P. semiovatus* (FJAU78355). (**F**) *P. bisporus* (FJAU78362); (**G**–**L**) Basidiospores: (**G**) *P. ovinus* (FJAU78336). (**H**) *P. latifolius* (FJAU78340). (**I**) *P. bambusicola* (FJAU78368). (**J**) *P. praecox* (FJAU78357). (**K**) *P. foenisecii* (FJAU78361). (**L**) *P. rhombispermus* (FJAU78367). Scale bars: (**A**–**I**) = 10 μm; (**J**–**L**) = 2 μm.

Based on scanning electron microscopy (SEM) observations of the spore ornamentation of *Panaeolus rhombispermus* and multi-locus phylogenetic analyses, we regard *P. rhombispermus* as distinct from other *Panaeolus* species (Figure 8). Considering that the type species of *Crucispora*, *Crucispora naucorioides* E. Horak, also possesses cruciform spores, we thus further propose to reduce *Crucispora* to a subgenus under *Panaeolus*. Previous work by Ostuni et al. [14] proposed transferring *C. rhombispermus* to the genus *Panaeolus*, arguing that its hymeniform pileipellis, mottled gills, and dark brown spores with a germ pore align with characteristics of *Panaeolus*. They further suggested that the distinctive cruciform spore shape of *P. rhombispermus* represents merely an extreme morphological variant within the known spore morphology range of *Panaeolus*, noting that a similar, albeit less pronounced, shape occurs in spores of *P. mexicanus* (Guzmán) Voto & Angelini. However, our observations indicate that the spore morphology and surface ornamentation of *P. rhombispermus* (Figure 8L) are fundamentally distinct from those of all known *Panaeolus* species. This is corroborated by light microscopy images from Chou et al. [40], which show pronounced spore surface ornamentation in *P. rhombispermus*, whereas spores of *P. mexicanus* appear smooth [38]. Furthermore, *P. mexicanus* possesses thick-walled pleurocystidia, a trait shared with other members of Clade V (*Panaeolus* subg. *Bresadolomyces*, including *P. bisporus* and *P. cyanescens*), while both currently recognized species of *Panaeolus* subg. *Crucispora* lack pleurocystidia. We note that in the original publications of the two *Crucispora* species, their spores were consistently described as “rhomboid” and “smooth”, with corresponding illustrations also depicting a smooth surface [12,13]. This conclusion, however, may have been constrained by the resolution limits of the microscopes available at the time, which likely prevented accurate recognition of the spore wall ornamentation. It should be emphasized that the rhomboid shape of the spores remains an accurate and stable diagnostic feature.

Our phylogenetic analysis reveals that *Panaeolus bambusicola* (FJAU78368, FJAU78369) and *P. sylvaticus* (ANGE1393) each form independent lineages, designated as Clade IV and Clade II, respectively, and neither is assigned to an existing subgenus. *P. bambusicola* is distinguished by a persistently pruinose pileus and broadly limoniform, relatively small basidiospores. Nevertheless, because its clade currently comprises only this species, we have not proposed a subgeneric placement for it. Conversely, the phylogenetic position of *P. sylvaticus* remains unstable, likely due to the current limitation to ITS sequences and a lack of additional sequence data for robust support. We expect that the future discovery of more *Panaeolus* species and the sharing of related molecular sequences will facilitate a more robust and stable subdivision of the genus.

## Figures and Tables

**Table 1:** Information of DNA sequences used in the phylogenetic analyses. Sequences newly generated in this study are shown in bold. “–” means data not available. Bold capital letters after voucher or strain represent Holotype “T”, Paratypes “P”.

Taxon	Voucher/Strain	Origin	ITS	nrLSU	*tef1*	*rpb2*	References
*Bolbitius coprophilus*	LE18599	Russia	KR425526	KR425556	–	–	[33]
*Conocybe muscicola*	HMJAU64939 **T**	China	OQ758113	OQ758223	OQ758309	–	[34]
***P. acuminatus***	**FJAU78351**	**China**	**PX868543**	**PX868580**	–	–	**This study**
*P. acuminatus*	CBS 270.47	Not indicated	MH856251	MH867783	–	–	[9]
*P. acuminatus*	KA16-1041	Kyrgyzstan	MK351680	–	–	–	[35]
*P. acuminatus*	X540	Czech Republic	MW352021	–	–	–	[35]
*P. acuminatus*	LUGO:ECC17111604	Spain	MW376698	–	–	–	[36]
*P. acuminatus*	SGL09	Not indicated	OR035540	–	–	–	[35]
*P. acuminatus*	AMB 20066	Italy	PP447481	PP447517	–	–	[37]
***P. alcis***	**FJAU78331**	**China**	**PX868551**	**PX868579**	**PX857983**	**PX852305**	**This study**
***P. alcis***	**FJAU78332**	**China**	**PX868540**	–	–	–	**This study**
*P. alcis*	SAT-14-239-20	USA(Alaska)	MW597122	–	–	–	[35]
***P. antillarum***	**FJAU78352**	**China**	**PX868546**	**PX868578**	**PX857978**	–	**This study**
***P. antillarum***	**FJAU78353**	**China**	**PX868537**	**PX868562**	**PX893654**	**PX852306**	**This study**
*P. antillarum*	UOC KAUNP MK62	Sri Lanka	KP764810	–	–	–	[35]
*P. antillarum*	UOC KAUNP K01	Sri Lanka	KR867660	–	–	–	[35]
*P. antillarum*	SFSU:DED 7874	Thailand	MF497585	–	–	–	[35]
*P. atrobalteatus*	jlh7468-14	USA	PP808684	–	–	–	–
*P. axfordii*	MFLU 19-2367 **T**	China	NR169700	–	–	–	[19]
***P. bambusicola***	**FJAU78368 T**	**China**	**PX868530**	**PX868563**	**PX893655**	**PX852307**	**This study**
***P. bambusicola***	**FJAU78369**	**China**	**PX868522**	–	–	–	**This study**
***P. bisporus***	**FJAU78362**	**China**	**PX868547**	**PX868577**	–	–	**This study**
***P. bisporus***	**FJAU78363**	**China**	**PX868550**	–	–	–	**This study**
***P. bisporus***	**FJAU78365**	**China**	**PX868532**	**PX868561**	–	–	**This study**
*P. bisporus*	HYW197	China	OR035518	–	–	–	[35]
*P. cambodginiensis*	NBRC-30222	Japan	AB158633	–	–	–	[35]
*P. castaneifolius*	Mushroom Observer 90428	USA	KX010428	–	–	–	[35]
*P. chlorocystis*	FMS iNaturalist # 193561740	USA	PQ678531	–	–	–	–
***P. cinctulus***	**FJAU78344**	**China**	**PX868557**	**PX868570**	**PX857981**	**PX852303**	**This study**
***P. cinctulus***	**FJAU78345**	**China**	**PX868538**	–	**PX893653**	**PX852304**	**This study**
*P. cinctulus*	xsd08077	Not indicated	FJ478119	–	–	–	[37]
*P. cinctulus*	CBS 326.34	Not indicated	MH855550	MH867055	–	–	[37]
*P. cinctulus*	CBS 328.34	Not indicated	MH855552	MH867057	–	–	[37]
*P. cinctulus*	NX180911-04		MN960188	–	–	–	[37]
*P. cinctulus*	MCVE 1084	Italy	PP447482	PP447518	–	–	[37]
*P. cinctulus*	Gerhardt 83052 (B)	Germany	PP447483	PP447521	–	–	[37]
***P. cyanescens***	**FJAU78366**	**Timor-Leste**	**PX868520**	–	–	–	**This study**
*P. cyanescens*	taxon:181874	Not indicated	HM035084	HM035084	–	–	[37]
*P. cyanescens*	BP1	India	MK855515	–	–	–	[37]
*P. cyanescens*	FS1	India	MK855516	–	–	–	[37]
*P. cyanescens*	AMB 20070	Italy	PP447484	PP447522	–	–	[37]
*P. cyanescens*	0709305/JG	France	PP447485	PP447523	–	–	[37]
*P. desertorum*	SZMC-NL-1863	Hungary	JX968154	JX968271	JX968387	–	[8]
*P. desertorum*	LE 2865	Uzbekistan	MH055383	–	–		[35]
*P. desertorum*	LE 2864	Uzbekistan	MH055384	–	–	–	[35]
*P. desertorum*	AH 9993 **P**	Spain	MK397543	MK397561	–	–	[35]
*P. desertorum*	LE313090	Russia	MK397566	MK397591	–	–	[37]
*P. desertorum*	GB-0073426	Hungary	PP447486	PP447524	–	–	[37]
*P. detriticola*	PERTH 08944954 **T**	Australia	NR199086	MT571659	–	–	–
*P. dunensis*	AMB 20210	Italy	PP447489	PP447527	–	–	[37]
*P. dunensis*	AMB 20211	Italy	PP447490	–	–	–	[37]
*P. fimicola*	MCVE 4084	Italy	JF908518	–	–	–	[37]
*P. fimicola*	CBS 251.37	Not indicated	MH855904	MH867411	–	–	[37]
*P. fimicola*	Gerhardt 75349 (B)	Germany	PP447492	PP447529	–	–	[37]
***P. foenisecii***	**FJAU78358**	**China**	**PX868536**	–	–	–	**This study**
***P. foenisecii***	**FJAU78359**	**China**	**PX868542**	–	–	–	**This study**
***P. foenisecii***	**FJAU78360**	**China**	**PX868539**	**PX868560**	**PX893657**	**PX852302**	**This study**
***P. foenisecii***	**FJAU78361**	**China**	**PX868554**	–	–	**PX852298**	**This study**
*P. foenisecii*	6643	Italy	JF908520	–	–	–	[37]
*P. foenisecii*	ubc F32593	Canada	MG969989	–	–	–	[37]
*P. foenisecii*	CBS 142.40	Not indicated	MH856067	MH867557	–	–	[37]
*P. foenisecii*	AMB 20071	Italy	PP447493	PP447530	–	–	[37]
*P. foenisecii*	AMB 20072	Italy	PP447494	PP447531	–	–	[37]
***P. fraxinophilus***	**FJAU78346**	**China**	**PX868544**	**PX868575**	**PX857980**	**PX852308**	**This study**
*P. fraxinophilus*	MushroomObserver.org/455364	USA	OL629088	–	–	–	[35]
*P. fraxinophilus*	OMDL iNat # 170758482	USA	OR987324	–	–	–	[35]
***P. latifolius***	**FJAU78340 T**	**China**	**PX868523**	–	–	–	**This study**
***P. latifolius***	**FJAU78341**	**China**	**PX868533**	**PX868565**	–	–	**This study**
*P. mediterraneus*	AMB 20074	Italy	PP447496		–	–	[37]
*P. mediterraneus*	AMB 20075	Italy	PP447497	PP447533	–	–	[37]
*P. mexicanus*	ANGE1557	Dominican Republic	MZ856314	–	OK546186		[38]
*P. olivaceus*	MushroomObserver.org/158389	USA	MF629829	–	–	–	[35]
*P. olivaceus*	MushroomObserver.org/89608	USA	MH285992	–	–	–	[35]
*P. olivaceus*	iNAT:100066497	USA	ON314881	–	–	–	[37]
*P. olivaceus*	AMB 20076	Italy	PP447498	PP447534	–	–	[37]
***P. ovinus***	**FJAU78335**	**China**	**PX868559**	–	–	–	**This study**
***P. ovinus***	**FJAU78336 T**	**China**	**PX868534**	**PX868566**	**PX857973**	**PX852299**	**This study**
***P. ovinus***	**FJAU78337**	**China**	**PX868535**	**PX868567**	**PX857974**	**PX852297**	**This study**
*P. pantropicalis*	JBSD 130972 **T**	Dominican Republic	PP590037	–	–	–	[35]
*P. pantropicalis*	PERTH 09605894 **P**	Australia	PP590039	–	–	–	[35]
***P. papilionaceus***	**FJAU78333**	**China**	**PX868549**	**PX868573**	**PX857972**	**PX852300**	**This study**
***P. papilionaceus***	**FJAU78334**	**China**	**PX868525**	–	–	–	**This study**
*P. papilionaceus*	AFTOL-ID 1499	Not indicated	DQ182503	DQ470817	–	–	[37]
*P. papilionaceus*	CBS 582.79	Not indicated	HM035081	HM035081	–	–	[37]
***P. parvisporus***	**FJAU78330**	**China**	**PX868556**	–	–	–	**This study**
*P. parvisporus*	MushroomObserver.org/312079	USA	MH101639	–	–	–	[35]
*P. plantaginiformis*	LE 2867	Uzbekistan	MK397575	MK397597	–	–	[9]
*P. plantaginiformis*	LE 2870	Uzbekistan	MK397576	MK397598	–	–	[9]
*P. plantaginiformis*	LE 2862	Russia	MK397577	MK397599	–	–	[9]
*P. plantaginiformis*	TAAM120547	Uzbekistan	PP447502	PP447537	–	–	[37]
*P. plantaginiformis*	TAAM120647	Uzbekistan	PP447503	–	–	–	[37]
***P. praecox***	**FJAU78356**	**China**	**PX868528**	**PX868568**	**PX857975**	**PX852309**	**This study**
***P. praecox***	**FJAU78357 T**	**China**	**PX868558**	**PX868569**	**PX857976**	–	**This study**
*P. punjabensis*	LAH36793 **T**	Pakistan	MZ265143	ON116490	–	–	[39]
*P. punjabensis*	LAH36794	Pakistan	MZ823627	ON116492	–	–	[39]
*P. retirugis*	CBS 274.47	France	MH856255	MH867787	–	–	[37]
***P. rhombispermus***	**FJAU78367**	**China**	**PX868531**	**PX868564**	**PX926009**	**PX926010**	**This study**
*P. rhombispermus*	CWN 11502	China	MZ782082	MZ781504	–	–	[40]
***P. rickenii***	**FJAU78348**	**China**	**PX868548**	**PX868572**	**PX857979**	**PX852296**	**This study**
***P. rickenii***	**FJAU78349**	**China**	**PX868541**	**PX868571**	–	–	**This study**
*P. rickenii*	HMAS 290093	China	MK966648	–	–	–	–
*P. rickenii*	HMAS 290109	China	MK966649	–	–	–	–
***P. semiovatus***	**FJAU78354**	**China**	**PX868526**	–	–	–	**This study**
***P. semiovatus***	**FJAU78355**	**China**	**PX868524**	–	–	–	**This study**
*P. semiovatus*	MCVE 21188	Italy	JF908515	–	–	–	[37]
*P. semiovatus*	CBS 276.39	Not indicated	MH856012	–	–	–	[35]
*P. semiovatus*	Mushroom6	China	MT451924	–	–	–	[35]
*P. semiovatus*	AMB 20084	Italy	PP447509	PP447542	–	–	[37]
*P. semiovatus*	AMB 20082	Italy	PP447511	PP447541	–	–	[37]
***Panaeolus* sp.**	**FJAU78338**	**China**	**PX868555**	–	**PX893656**	–	**This study**
***Panaeolus* sp.**	**FJAU78339**	**China**	**PX868545**	–	–	–	**This study**
***Panaeolus* sp.**	**FJAU78350**	**China**	**PX868521**	–	–	–	**This study**
***Panaeolus* sp.**	**FJAU78364**	**China**	**PX868553**	–	–	–	**This study**
***P. subbalteatus***	**FJAU78342**	**China**	**PX868552**	**PX868576**	**PX857977**	**PX852301**	**This study**
***P. subbalteatus***	**FJAU78343**	**China**	**PX868527**	–	–	–	**This study**
*P. subbalteatus*	CBS 327.34	USA	MH855551	MH867056	–	–	[37]
*P. subbalteatus*	HFJAU-ND146	China	MN622762	–	–	–	[35]
*P. sylvaticus*	ANGE1393	Dominican Republic	OQ311002	–	–	–	[35]
*P. tropicalis*	Not indicated	China	JF961377	–	–	–	–

## Data Availability

All the sequences have been deposited in GenBank (https://www.ncbi.nlm.nih.gov, accessed on 26 January 2026) and MycoBank (https://www.mycobank.org, accessed on 29 January 2026). The data presented in this study are deposited in the Zenodo repository, accession number https://doi.org/10.5281/zenodo.18431687, accessed on 30 January 2026.
